# International Network for Comparison of HIV Neutralization Assays: The NeutNet Report II

**DOI:** 10.1371/journal.pone.0036438

**Published:** 2012-05-09

**Authors:** Leo Heyndrickx, Alan Heath, Enas Sheik-Khalil, Jose Alcami, Vera Bongertz, Marianne Jansson, Mauro Malnati, David Montefiori, Christiane Moog, Lynn Morris, Saladin Osmanov, Victoria Polonis, Meghna Ramaswamy, Quentin Sattentau, Monica Tolazzi, Hanneke Schuitemaker, Betty Willems, Terri Wrin, Eva Maria Fenyö, Gabriella Scarlatti

**Affiliations:** 1 Unidad de Immunopatologia del SIDA, Instituto de Salud Carlos III, Madrid, Spain; 2 Laboratory of AIDS and Molecular Immunology, Fundação Oswaldo Cruz, Rio de Janeiro, Brazil; 3 Department of Laboratory Medicine, University of Lund, Lund, Sweden; 4 National Institute for Biological Standards and Control, Potters Bar, Hertfordshire, United Kingdom; 5 Virology Unit, Biomedical Department, Institute of Tropical Medicine, Antwerp, Belgium; 6 Department of Microbiology, Tumor and Cell Biology, Karolinska Institutet, Stockholm, Sweden; 7 Unit of Human Virology, San Raffaele Scientific Institute, Milan, Italy; 8 Duke University Medical Center, Durham, North Carolina, United States of America; 9 Pathogénie des infections persistantes, University Louis Pasteur, Strasbourg, France; 10 National Institute for Communicable Diseases, Johannesburg, South Africa; 11 WHO-UNAIDS HIV Vaccine Initiative, World Health Organization, Geneva, Switzerland; 12 Department of Vaccine Research, Henry Jackson Foundation for the Advancement of Military Medicine, Rockville, Maryland, United States of America; 13 The Sir William Dunn School of Pathology, The University of Oxford, Oxford, United Kingdom; 14 Viral Evolution and Transmission Unit, San Raffaele Scientific Institute, Milan, Italy; 15 Department of Experimental Immunology, Academic Medical Center at the University of Amsterdam, Amsterdam, The Netherlands; 16 Monogram Biosciences, San Francisco, California, United States of America; Shanghai Medical College, Fudan University, China

## Abstract

**Background:**

Neutralizing antibodies provide markers for vaccine-induced protective immunity in many viral infections. By analogy, HIV-1 neutralizing antibodies induced by immunization may well predict vaccine effectiveness. Assessment of neutralizing antibodies is therefore of primary importance, but is hampered by the fact that we do not know which assay(s) can provide measures of protective immunity. An international collaboration (NeutNet) involving 18 different laboratories previously compared different assays using monoclonal antibodies (mAbs) and soluble CD4 (Phase I study).

**Methods:**

In the present study (Phase II), polyclonal reagents were evaluated by 13 laboratories. Each laboratory evaluated nine plasmas against an 8 virus panel representing different genetic subtypes and phenotypes. TriMab, a mixture of three mAbs, was used as a positive control allowing comparison of the results with Phase I in a total of nine different assays. The assays used either uncloned virus produced in peripheral blood mononuclear cells (PBMCs) (Virus Infectivity Assays, VIA), or Env (gp160)-pseudotyped viruses (pseudoviruses, PSV) produced in HEK293T cells from molecular clones or from uncloned virus. Target cells included PBMC and genetically engineered cell lines in either single- or multiple-cycle infection format. Infection was quantified by using a range of assay read-outs including extra- or intra-cellular p24 antigen detection, luciferase, beta-galactosidase or green fluorescent protein (GFP) reporter gene expression.

**Findings:**

Using TriMab, results of Phase I and Phase II were generally in agreement for six of the eight viruses tested and confirmed that the PSV assay is more sensitive than PBMC (p = 0.014). Comparisons with the polyclonal reagents showed that sensitivities were dependent on both virus and plasma.

**Conclusions:**

Here we further demonstrate clear differences in assay sensitivities that were dependent on both the neutralizing reagent and the virus. Consistent with the Phase I study, we recommend parallel use of PSV and VIA for vaccine evaluation.

## Introduction

Interest in HIV neutralization as a correlate of immune protection has been inconsistent over the years. Initial vaccine trials in the early 1990s were discouraging after discovering that neutralizing antibodies, if elicited at all, had narrow specificity, and were only directed to the virus strain included in the vaccine [1], [2], [3], [4], [5], [6]. Cell-mediated immunity (CMI) came into focus, but subsequent vaccine trials eliciting CMI showed no greater success in protection from HIV infection [7], [8]. Early in the 2000s interest turned back to neutralization, and the idea that a vaccine should aim to elicit both humoral and cellular immune responses was put forward [9]. It was felt that by mounting a broad neutralizing antibody response the immune response might overcome virus variation [10], [11], [12]. In view of this development, standardization of evaluation of neutralizing activity became an important issue [13], [14], [15], [16].

In 2004, a group of 18 laboratories, performing a range of different techniques to measure neutralizing antibodies, was assembled within the framework of an EC-sponsored international collaborative study, called NeutNet. The group aimed at the standardization of HIV-1 neutralization assays to be used in vaccine research and clinical vaccine trials, by testing different monoclonal antibodies (mAbs) and soluble (s)CD4 against 11 HIV-1 isolates and their clonal derivatives in 10 different neutralization assays. The NeutNet Phase I study showed that: 1) in general, PSV assays were more sensitive than VIA; 2) variation was dependent on both the reagent (in this case mAbs and sCD4) and the virus used; 3) the apparent larger variation in the PBMC assays was probably due to different operating procedures in the participating laboratories. It was concluded that no single assay was capable of detecting the entire spectrum of antibody neutralizing activities. Since it is not known which *in vitro* assay correlates with *in vivo* protection, the use of a range of assays was recommended [17].

In 2008, NeutNet continued its activity within the EUROPRISE network of Excellence by comparing neutralization assays with polyclonal reagents, carefully selected for the purpose and centrally distributed to all partners. The polyclonal reagents were tested against eight viruses, selected from the previous Phase I virus panel, in the different neutralization assays. The results of the network study, now comprised of nine different assays are presented herein.

## Methods

### Neutralization Assays

**Figure 1:**
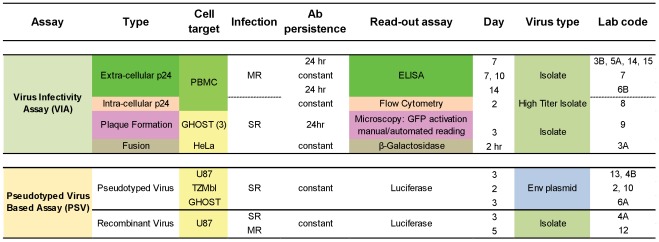
Neutralization assays and their characteristics. Cell target: PBMC, peripheral blood mononuclear cells; the cell lines GHOST, U87 and HeLa are stably transfected with CD4 and CCR5 or CXCR4. MR, multiple round infection; SR, single round infection. The fusion assay is limited to cell surface-viral envelope interaction. Ab persistence: time of incubation of the inhibitory reagent with virus and cells before washout. Day: time of read-out, numbers indicate days; hr, hours. Env plasmid, Env expression plasmids obtained through NIBSC.

The methodologies used in this study were previously published [17] and are available on the EUROPRISE website (www.europrise.org). Briefly, two kinds of assays were performed: (1) Virus Infectivity Assays (VIA) using replicating viruses and Env (gp160)-pseudotyped virus (PSV) assays (Figure 1). In the first type of assay, partners 3B, 5A, 6B, 7, 8, 14 and 15 used peripheral blood mononuclear cells (PBMC) as target cells, while lab 9 and 3A used established cell lines [18], [19], [20], [21], [22]. PBMCs were isolated from buffy coats from HIV-negative blood donors as previously described, detailed protocols are available on the EUROPRISE website (www.europrise.org). Partner 9 performed a plaque reduction assay using GHOST(3) cells and partner 3A used a fusion assay with HeLa cells [17], [23], [24], [25], [26]. Both cell lines were engineered to express CD4 and coreceptors for HIV. In all labs using VIA, except lab 3A, 8 and 9, assays were characterized by multiple rounds of infection. The PSV assays performed by partners 2, 4A, 4B, 6A, 10 and 13 were single cycle assays [27], [28], [29]. The PSV assay performed by partner 12, was a multiple cycle infection assay [30], [31]. Two readouts were used for the plaque reduction assay on GHOST(3) cells, both exploiting activation of the gene encoding the green fluorescence protein (GFP) upon HIV infection. Plaques were either manually counted by microscopic reading, as previously described, or by a newly developed automated microscopy reading platform followed by image analysis using the CellProfiler software version r10997 [32] (www.cellprofiler.org ). The pipeline used will be described in detail separately (Sheik-Khalil, manuscript in preparation).

### Inhibitory Reagents

All reagents were distributed by the Centre for AIDS Reagents (CFAR) NIBSC, UK. TriMab, an equal mixture of three mAbs IgG1b12, 2G12 and 2F5 was prepared by CFAR and was used as positive control in each experiment and for comparison with Phase I results. In order to have reagents with both high and low neutralizing titres in both types of assays, 19 HIV-1-positive plasma obtained from Zeptometrix Corporation (USA) were pre-screened in the recombinant virus assay against a total of 14 viruses, of which 9 were available both as culture supernatants (CC) as well as DNA. Three of the plasma with the highest neutralization scores, defined as the proportion of tested viruses neutralized, were selected for inclusion in the final Phase II panel. Similarly, an additional five plasma samples were selected out of 57 HIV-1-positive samples of which 40 samples were previously collected in Uganda and 17 were provided by the Blood Transfusion Service (BTS), UK. The selected five samples neutralized the highest number of viruses (23, 23, 20, 19 and 27 of the possible 27 positive reactions). Full details of this selection are available as supplemental information (Table S1). An HIV negative plasma (source: BTS, UK) was also included in the final panel as negative control. The final selection of reagents is shown in Table 1. All reagents were deposited with CFAR at NIBSC for central storage and further use.

**Table 1:** List of inhibitory reagents selected.

ARP number	Anti-HIV status	Characteristics	Lot n°	Donor Origin
**515**	Positive	US Blood Donor	01654	Zeptometrix Inc, USA
**516**	Positive	US Blood Donor	01661	Zeptometrix Inc, USA
**517**	Positive	US Blood Donor	01684	Zeptometrix Inc, USA
**518**	Positive	African Donor	G0724067163956	Dr. D Howell, BTS, UK (NIBSC)
**519**	Positive	African Donor	G0746057158836	Dr. D Howell, BTS, UK (NIBSC)
**520**	Positive	African Donor	G0746067341811	Dr. D Howell, BTS, UK (NIBSC)
**521**	Positive	African Donor	543801.2	BTS, UK (NIBSC)
**522**	Positive	Subtype F; Brazil	100791915	Dr. E Sabino, Sao Paulo, Brazil (NIBSC)
**523**	Negative	HIV negative sample	G151703582418C	BTS, UK (NIBSC)
**513**	Positive	HIVIG 1031	HIV-IgG 990909	Dr. B Warren, Sweden
**3240.1**	Positive	TriMab (1 mg/ml)	20.11.06	Dr. Katinger, Austria Dr. Burton, USA (NIBSC)

Two series of neutralization assays were run. In the first assay series TriMab was used at an initial concentration of 25 µg/ml followed by five 4-fold dilutions. For all plasma, including the HIV-negative plasma, a starting dilution of 1/20 followed by four 4-fold dilutions was used. In the second series of assays, the highest starting concentration/dilution of the inhibitor used was around the end-point obtained in the first series followed by four 2-fold dilutions, allowing a more precise calculation of inhibitory concentrations.

The 50% inhibitory concentrations (IC50) were expressed in µg/ml for TriMab or as the reciprocal serum dilution resulting in 50% reduction of virus growth. Final concentrations of the inhibitor were calculated from virus-inhibitory reagent mixtures, before addition of cells.

### Viruses

**Figure 2:**
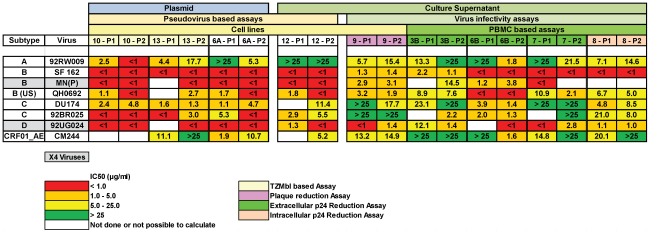
Mean inhibitory concentration (IC) 50 values for duplicate assays performed with TriMab and virus as indicated in the NeutNet Phase I (P1) and Phase II (P2) study. The cells are colour coded: green, poor or no neutralization, IC50>25 µg/ml; yellow, IC50 5–25 µg/ml; orange, IC50 1–5 µg/ml; red, IC50<1 µg/ml; white, no results available. Assays are grouped on the basis of several criteria: (1) the use of plasmids or culture supernatants as a source of HIV-1; (2) fusion based assays or infection based assays, either with pseudotyped virus or replication competent virus; and (3) the use of cell lines or PBMC. Laboratories performing the assays are numbered (see Figure 1 for reference) and colour coded: blue, TZMbl assay or PSV/plasmid assays; green, PBMC assays using extracellular p24 as readout; pink, plaque reduction assay. In the listing of viruses, to the left, the cells of X4 viruses are labelled grey, the cells of R5 viruses are white.

Eight HIV-1 isolates (Figure 2) selected from the panel of 12 used in the NeutNet phase I study [17] and/or their clonal derivatives were used. The viruses chosen represented different HIV-1 subtypes, varying neutralization sensitivity and coreceptor usage. All viruses were prepared and supplied to each participant by CFAR at NIBSC thereby ensuring that all the laboratories had a common starting material. Each participating laboratory subsequently expanded virus stocks and plasmids needed and performed titrations before use. In contrast to the NeutNet phase I study, all laboratories using a PSV assay received HEK293T cells provided by Lynn Morris through CFAR (originating from David Montefiori’s lab) to prepare the pseudovirus stocks, thereby excluding potential differences due to the source of cells used for production.

### Statistical Analysis

Analysis was based on the raw assay data returned by participating laboratories. Each laboratory was requested to perform the assays twice according to their standard protocol, with all dilutions tested at least in duplicates. The 50%, 75% and 90% inhibitory concentrations (IC50, IC75 and IC90) were calculated with a linear interpolation method, using the mean of duplicate responses, as previously published [17]. Briefly, the assay readout equivalent to the IC50 was calculated as half the assay readout with no antibody present (similarly for IC75 and IC90). The dilution interval containing the IC50 was identified, with assay readout for adjacent dilutions being above and below the 50% readout. The assay readouts for the dilutions above and below the IC50 were joined with a straight line, and plotted against the log concentration of antibody. Where the IC value was outside the range of concentrations tested, it was recorded as either greater than the highest concentration used, or less than the lowest concentration, as appropriate. Where the assay data were variable, and the observed dose-response crossed the relevant percentage inhibition level (e.g. 50% inhibition for IC50) more than once, no IC value was calculated. Absence of a calculated IC value may therefore be due to a laboratory not testing a particular combination of virus and antibody, or to the resulting assay data being too variable to allow a calculation. The variable data quality precluded the use of more sophisticated curve-fitting models for calculation of IC values.

The geometric mean IC50s of laboratories performing PSV or PBMC assays were calculated for each virus. Differences in sensitivity between the PSV or PBMC assay were assessed by calculating the fold-difference in geometric mean IC50 for each virus, and performing a Wilcoxon 1-sample test, comparing the median fold-difference to 1.0 (representing equivalent sensitivity). This was done for TriMab, and for the IC50s averaged across the positive plasmas ARP515– ARP522.

## Results

### Comparison of NeutNet Phase I and II TriMab Neutralization

**Figure 3:**
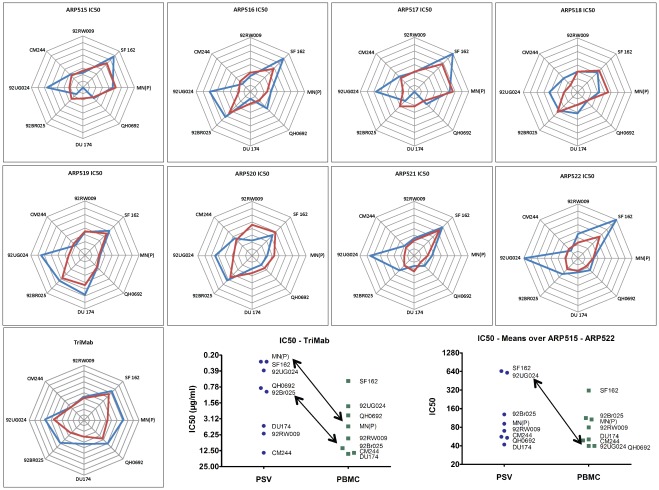
Comparison of PSV and VI assays across viruses. (A) circular “radar” plots. Lines from the centre represent an axis for each virus. The geometric mean IC value for PSV (blue lines) and PBMC (red lines) against each virus is plotted, and the points joined. The scale is set such that the centre represents no neutralization and the concentric grid-lines are 2-fold dilution steps moving out to highest neutralization at the edge. (B) and (C) Ranking of viruses for relative sensitivity to neutralization was done by calculating geometric mean IC50s across laboratories (grouping PSV and PBMC separately). (B) Ranking by TriMab and (C) ranking by plasma (means over ARP515-522). The scale is set such that the most neutralization sensitive viruses are at the top.

Since the aim of this study was to compare the performance of a wide variety of HIV-1 neutralization assays as performed in different laboratories and since TriMab was included in both Phase I and II, it allowed comparison of IC50s both within the same laboratory and between laboratories. Results from nine laboratories, participating in both Phase I and Phase II studies showed that results were generally in agreement with six of the eight viruses tested (Figure 2). In phase II, two viruses (92RW009 and CM244) showed a mean variation >3-fold, for all other viruses this was less than 3-fold. In addition, the mean IC50s obtained in the PSV assays were lower, meaning more sensitive, than in PBMC (p = 0.014) (Table 2, Figure 3b).

**Table 2:** Inter-Laboratory comparisons IC50 values.

	Pseudovirus Based Assays										PBMC Assays										Fusion		Plaque reduction	
	Labs 6A,10,12,13										Labs 3B,6B,7,8,14,15										Lab 3A		Lab 9	
**TRIMAB**																								
Virus	N lab		Mean		Min		Max		Fold		N lab		Mean		Min		Max		Fold		Mean		Manual reading	Automated reading
92RW009	4		5,9		0,3		38,8		114,5		6		7,3		0,6		>50		90,7		26,1		15,4	4,8
SF 162	4		0,3		<0,2		0,5		2,5		6		0,6		<0,2		1,8		9,2		1,5		1,4	1,8
*MN(P)*	4		0,3		<0,2		0,7		3,5		4		4,3		1,1		14,5		13		1,9		3,1	5,2
QH0692	4		0,8		0,4		2,7		7,5		5		2,7		0,3		7,6		27,9		10,7		1,9	2,8
DU174	4		4,2		1,3		11,4		9,1		5		14,3		1,4		>50		35,2				17,7	15,4
92BR025	4		1		<0,2		5,5		27,4		5		11,2		1,3		>50		37,5		32,7		28,1	14,8
*92UG024*	4		0,4		<0,2		0,8		4		6		1,8		0,6		8,2		14,5		3,6		1,4	1,5
CM244	3		13,7		5,2		45,9		8,8		6		13,7		1		>50		50,5		11,9		14,9	8,8
**ARP 515**																								
Virus	N lab		Mean		Min		Max		Fold		N lab		Mean		Min		Max		Fold		Mean		Manual reading	Automated reading
92RW009	4		48		<20		542		27,1		6		61		<20		410		20,5		47		392	1544
SF 162	4		1070		471		>1280		2,7		6		386		197		718		3,6		54		130	91
*MN(P)*	4		252		115		477		4,2		3		340		90		937		10,4		221		497	544
QH0692	4		52		22		92		4,1		5		46		<20		320		16		<20		82	62
DU174	4		<20		<20		23		1,2		5		33		<20		179		8,9				22	<20
92BR025	4		28		<20		54		2,7		5		58		40		79		2		53		31	<20
*92UG024*	4		478		<20		>1280		64		6		43		<20		121		6,1		36		40	58
CM244	3		69		47		129		2,7		6		58		<20		512		25,6		63		31	61
**ARP 516**																								
Virus	N lab		Mean		Min		Max		Fold		N lab		Mean		Min		Max		Fold		Mean		Manual reading	Automated reading
92RW009	4		52		<20		1054		52,7		6		70		34		220		6,5		55		32	461
SF 162	4		1209		726		>1280		1,8		6		281		44		962		21,8		109		186	255
*MN(P)*	4		81		50		281		5,7		4		55		<20		130		6,5		98		<20	<20
QH0692	4		113		53		925		17,4		5		35		<20		93		4,7		<20		<20	<20
DU174	4		20		<20		29		1,5		5		33		<20		262		13,1				<20	23
92BR025	4		374		286		536		1,9		5		216		45		500		11,1		65		<20	96
*92UG024*	4		659		45		>1280		28,3		6		32		<20		130		6,5		45		21	363
CM244	3		32		28		39		1,4		6		44		<20		160		8		114		34	23
**ARP 517**																								
Virus	N lab		Mean		Min		Max		Fold		N lab		Mean		Min		Max		Fold		Mean		Manual reading	Automated reading
92RW009	4		74		<20		936		46,8		6		81		28		350		12.3		<20		47	155
SF 162	4		>1280		475		>1280		2,7		6		555		263		1236		4.7		27		113	625
*MN(P)*	4		370		182		921		5,1		4		506		215		1427		6.0		140		249	421
QH0692	4		58		32		137		4,3		5		37		<20		62		3.1		<20		31	28
DU174	4		<20		<20		31		1,5		5		44		<20		497		24.9				<20	22
92BR025	4		38		24		71		2,9		5		82		<20		301		15.1		<20		22	22
*92UG024*	4		503		20		>1280		62,5		6		33		<20		160		8.0		<20		<20	79
CM244	3		92		69		137		2		6		73		<20		286		14.3		<20		34	63
**ARP 518**																								
Virus		N lab		Mean		Min		Max		Fold		N lab		Mean		Min		Max		Fold		Mean	Manual reading	Automated reading
92RW009		4		82		36		253		7		6		81		40		280		7		86	39	325
SF 162		4		187		119		293		2,5		6		232		52		1220		23,3		52	74	90
*MN(P)*		4		92		64		203		3,2		4		233		<20		>1280		64		86	32	116
QH0692		4		38		27		58		2,2		5		35		<20		117		5,8		<20	<20	<20
DU174		4		88		23		775		33		5		36		<20		151		7,6			<20	<20
92BR025		4		149		120		176		1,5		5		180		37		>1280		34,2		108	<20	81
*92UG024*		4		181		20		>1280		63,2		6		40		<20		79		4		71	<20	60
CM244		3		74		57		122		2,1		6		27		<20		71		3,6		164	<20	82
**ARP 519**																								
Virus		N lab		Mean		Min		Max		Fold		N lab		Mean		Min		Max		Fold		Mean	Manual reading	Automated reading
92RW009		4		108		35		1173		33,2		5		115		<20		252		12,6		85	54	327
SF 162		4		346		107		861		8,1		6		236		45		1280		28,7		46	243	747
*MN(P)*		4		46		23		70		3,1		4		42		<20		110		5,5		70	35	<20
QH0692		4		59		24		203		8,5		5		56		<20		181		9,1		<20	57	65
DU174		4		578		371		1114		3		5		220		160		320		2			640	2023
92BR025		4		418		157		1140		7,3		5		279		93		1810		13,7		150	320	280
*92UG024*		4		921		66		6788		19,4		6		54		<20		190		9,5		69	21	84
CM244		3		41		22		57		2,6		5		53		21		226		11		140	<20	24
**ARP 520**																								
Virus		N lab		Mean		Min		Max		Fold		N lab		Mean		Min		Max		Fold		Mean	Manual reading	Automated reading
92RW009		4		48		<20		995		49,8		5		232		60		>1280		21.3		54	166	149
SF 162		4		194		121		456		3,8		6		302		63		>1280		20.3		21	219	1072
*MN(P)*		4		46		<20		161		8,1		4		96		<20		865		43.3		53	<20	<20
QH0692		4		38		31		46		1,5		5		62		<20		301		15.1		<20	59	38
DU174		4		38		26		58		2,2		5		61		21		310		14.8			80	119
92BR025		4		367		205		858		4,2		5		240		130		345		2.7		77	356	628
*92UG024*		4		437		56		1031		18,6		5		65		<20		221		11.1		64	57	136
CM244		3		126		65		185		2,9		6		95		28		400		14.1		115	49	123
**ARP 521**																								
Virus		N lab		Mean		Min		Max		Fold		N lab		Mean		Min		Max		Fold		Mean	Manual reading	Automated reading
92RW009		4		59		<20		>1280		64		6		46		<20		304		15.2		22	21	<20
SF 162		4		589		301		>1280		4,3		6		393		60		>1280		21.2		29	98	42
*MN(P)*		4		64		28		178		6,3		4		40		<20		270		13.5		78	<20	<20
QH0692		4		46		24		114		4,8		5		26		<20		61		3.1		<20	27	<20
DU174		4		29		<20		89		4,4		5		52		<20		396		19.8			<20	28
92BR025		4		86		34		225		6,7		5		42		<20		330		16.5		28	<20	<20
*92UG024*		4		900		81		>1280		15,7		6		27		<20		194		9.7		<20	<20	32
CM244		3		40		23		94		4,1		6		33		<20		100		5.0		40	27	<20
**ARP 522**																								
Virus		N lab		Mean		Min		Max		Fold		N lab		Mean		Min		Max		Fold		Mean	Manual reading	Automated reading
92RW009		4		123		21		>1280		61,1		5		50		<20		99		5		68	23	<20
SF 162		4		>1280		682		>1280		1,9		5		239		50		991		19,7		88	<20	22
*MN(P)*		4		49		21		345		16,6		4		47		<20		615		30,7		152	<20	<20
QH0692		4		58		<20		109		5,5		5		33		<20		89		4,4		<20	<20	<20
DU174		4		42		23		100		4,4		5		36		20		101		5			69	198
92BR025		4		101		72		150		2,1		5		50		32		101		3,2		114	35	<20
*92UG024*		4		>1280		212		>1280		6,1		6		36		<20		272		13,6		89	<20	<20
CM244		3		29		20		40		2		6		39		<20		326		16,3		81	<20	20

Values of the IC50s are expressed as reciprocal dilutions for plasma and as µg/ml for TriMab. N lab; Number of laboratories involved.

### Neutralization with Polyclonal Reagents

Plasma from an individual who tested negative for HIV (ARP523) was included in all experiments. Results from six laboratories showed occasional low-level neutralization (detected at IC50 but not at IC75) with the HIV-negative plasma, while five laboratories tested negative with all viruses. The rare positive reactions were randomly distributed among viruses and included both types of assays (Figure S1).

The intra-laboratory consistency for repeat tests was assessed by comparing the calculated IC values (expressed as a fold-difference (maximum/minimum)) for the HIV-1 positive samples. Analysis was restricted to tests where it had been possible to calculate an IC value from the data for both tests, and the calculated IC values were within the dilution range used. There was reasonable intra-laboratory consistency with a mean difference between tests of less than 2-fold (Table S2a) for each IC value, and no significant differences between IC50, IC75 or IC90. The IC50 was selected for all subsequent analysis.

The calculations were repeated for laboratories using PSV and VIA (using PBMC) separately (Table S2b). The fold-differences between repeat tests for the PSV assays were lower than for the PBMC assays (around 1.7-fold and 2.0-fold respectively), indicating modestly better intra-laboratory consistency for the PSV assays.

### Comparison of Plasma Neutralizing Activities in PSV and PBMC Assays

The relative neutralization performance of the different assays across viruses at the IC50 levels, are shown in Table 2. For completeness the information at the IC75 and IC90 levels is shown in Table S3. Comparison of PSV (blue line) and PBMC (red line) is highlighted in circular “radar” plots (Figure 3A). The scale is set such that the centre represents no neutralization and the concentric grid-lines are 2-fold dilution steps moving out to highest neutralization at the edge. Equal IC’s against each virus would result in a circular pattern. However, the curves assume different shapes and the IC50 concentrations obtained in PBMC do not always show the same pattern as seen with PSV assays (Figure 3A).

Strikingly, the relative pattern of neutralization obtained in PSV and PBMC assays with different viruses varies for different plasmas. For example, 92UG024 and SF162 were more sensitive to neutralization by ARP522 in the PSV than VI assays. Conversely, 92RW009, SF162, MN(P) and QH0692 were better neutralized by ARP520 in the PBMC than the PSV assay. Taken together, there are substantial differences in neutralization of individual viruses by different plasma. Using TriMab higher sensitivity was observed in the PSV assays for all viruses except CM244 and 92RW009 where comparable IC50 were obtained for both PSV and PBMC assays. This allows ranking of viruses for relative sensitivity to neutralization by TriMab (Figure 3B) and plasma (means over ARP515-522) (Figure 3C). While SF162 was the most sensitive virus in both types of assays with both monoclonal and polyclonal reagents, MN(P) is on the top with TriMab in the PSV assay only. The primary virus 92UG024 had a similarly high sensitivity in both assays when neutralized by TriMab, but was sensitive to polyclonal antibodies in the PSV assay only. Comparison of IC50 values with TriMab between the two types of assay showed that the PSV assay was generally more sensitive than PBMC assay (Figure 3B). The differences in sensitivity were calculated (ratio of IC50 values for PSV and PBMC) for each virus, and there was a median 3.4-fold increase in sensitivity for PSV across viruses. This is significantly different from a median of 1.0 which would represent equivalent sensitivity for the assay methods (p = 0.014, Wilcoxon 1-sample test). However, with the polyclonal reagents the difference in sensitivity between PSV and PBMC was less pronounced with the exception of 92UG024. The median fold-increase was 1.1, which was not significantly different from 1.0. It has to be noted that this overall pattern is based on means across laboratories and plasma and it may therefore mask differences in individual lab results, or for different plasma.

### Comparison of Virus Sensitivities to Neutralization in the PSV and PBMC Assays

Differences in sensitivities of viruses to neutralization by the different plasmas was further analysed by using a separate radar plot for each virus with plasma around the circle. Again, the scales were adjusted such that no neutralization (IC50<20) is at the centre, and the outer ring is strong neutralization (IC50>1280). The concentric grid-lines are 2-fold dilution steps.

**Figure 4:**
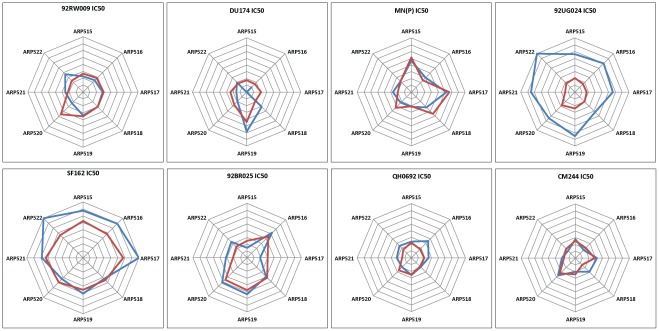
Comparison of PSV and VI assays across plasma by circular “radar” plots. The scales were adjusted such that no neutralization (IC50<20) is at the centre, and the outer ring is strong neutralization (IC50>1280). The concentric grid-lines are 2-fold dilution steps. Lines from the centre represent an axis for each plasma. The geometric mean IC value for PSV (blue lines) and PBMC (red lines) against each plasma is plotted, and the points joined.

Accordingly 92UG024 and SF162 have the highest IC50s (Figure 4), meaning high sensitivity to neutralization but only in the PSV assay. For 92UG024 it is notable that the PSV assays (blue line) were universally more sensitive than PBMC assays, but that was not the case for the other viruses. SF162 showed that PSV assays were a lot more sensitive for neutralization by ARP515, 516, 517 and 522, but not for 518, 519, 520 or 521. For 92RW009, ARP520 stands out as having more potent neutralizing activity in PBMC than PSV assays. This is all based on means across laboratories, so may still be masking individual differences in sensitivity between laboratories performing the same type of assay. In particular, we looked carefully at the outstanding sensitivity of 92UG024.

The IC50’s from lab 12 differed from those of the 3 other laboratories (6A, 10 and 13) performing PSV assays. Looking at the geometric mean across all eight plasma, the mean IC50 from laboratories 6A, 10 and 13 was 1284, compared to 45 from laboratory 12. This compares to a mean IC50 of 36 from the laboratories performing PBMC assays. The difference in IC50 between laboratory 12 (45) and the other PSV laboratories (1284) was highly significant (p<0.001, paired t-test) while the difference between lab 12 and the laboratories performing PBMC assays (36) was not (p = 0.29) (Figure S3). A possible explanation might be that lab 12 used a pool of amplified clones starting from viral supernatant, whereas a single clone was used by the three other laboratories. A pool of amplified clones might better represent the quasi-species present in a virus as compared to one single clone. Partial sequence analysis was done on both the virus culture supernatant and the plasmid used for the pseudovirus production. Overall 99% homology (amino acid level) was found for near the complete env gene (2190bp). Amino acid differences were found in the C2 (at position 204 A or E) and in gp41 (at position 845 T or A) respectively. Another 3 double amino acid populations were found in gp41 at positions 595, 732 and 734 (numbering according to HxB2) in the culture supernatant (Figure S2). We cannot exclude that the differences found between the culture supernatant and the PSV plasmid are responsible for the dramatic differences seen in neutralization sensitivity. This would require further analysis by use of site directed mutants, however this was outside the scope of our study. Another, maybe more important difference is that the PSV assay used by lab 12 is a multiple cycle assay as compared to a single cycle assay used by the 3 other laboratories performing the PSV assay. This further emphasizes the influence of the assay on the outcome of results.

### Evaluation of Neutralization by Plaque Reduction: Comparison of Manual Reading and Automated Image Analysis

**Figure 5:**
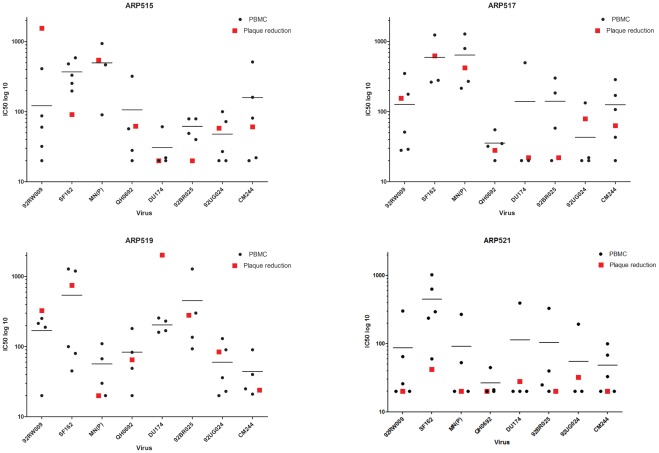
Comparison of PBMC assay with the automated readout of the plaque reduction assay. Plaques, identified as GFP-expressing cells, were evaluated by use of an AxioVision Z1 Microscope with automated reading platform. The 96-well plates were screened through with illumination time of 200 ms throughout experiments. To reduce auto fluorescence, medium was removed and PBS was gently added pre-microscopy. Plaque quantity was measured with CellProfiler software ( [32] (www.cellprofiler.org), version r10997. Image analysis was performed using fifteen 5× mosaic images per well. Results presented are the means of 2–3 experiments. Black dots, IC50 obtained by individual laboratories in the PBMC assay; red squares, IC50 obtained in the plaque reduction assay.

For these experiments, lab 9 used GHOST(3) cells and exploited activation of the green fluorescent protein (GFP) in HIV-infected cells [25], [33]. Cells showing green fluorescence were enumerated either visually or by use of an automated platform attached to an AxioVision Z1 microscope. Out of a total of 76 neutralization reactions 46 (61%) showed similar potency of neutralization between the two types of readouts (Table 2). In the remaining 30 reactions the potency of neutralization was different, such that automated reading gave stronger neutralization in 20 cases (67%). Comparison to the PBMC assays showed that in three virus-plasma combinations the plaque reduction (PR) assay yielded higher IC50 than the maximum IC50 obtained in the PBMC assay and in three other combinations IC50s were below that of the minimum value obtained in the PBMC assay. In all other cases the results of PR assay were within the range of min/max values of inter-lab variation for PMBC assays (Figure 5). Performance in the PR assay appeared to be random since no selectivity for a particular reagent or virus was observed. The results show that the previously standardized and validated PR assay can be subjected to automated reading allowing high-throughput application and further improvement of assay sensitivity. The PR assay has been repeatedly shown to be highly reproducible, sensitive and cheap [23], [24], [34], [35], [36]. It is now available with high throughput readout and could be considered as an alternative to the PBMC assay.

## Discussion

The primary aim of this study was to compare methods for the measurement of HIV-1 neutralizing antibodies in order to make recommendation for general use of one or two assays in research as well as in clinical trials of candidate HIV-1 vaccines. The results show that assay outcome is dependent on both the virus and the inhibitory reagent used, as well as the type of assay. This is in line with the observation made in the NeutNet Phase I study where monoclonal antibodies and soluble CD4 were used (www.europrise.org) [17]. The present study has extended this observation by including polyclonal reagents (plasma from HIV-1 infected individuals).

A comparison of the intra-laboratory consistency obtained when using either the IC50, IC75 or IC90 did not indicate that one had a significant advantage over the others, with all three giving mean differences between repeat tests of less than 2-fold. The IC50 was used in all subsequent analyses. In most of the assays 90% or even 75% of neutralization could not be achieved at the lowest plasma dilution (1:20) tested, and use of the IC50 levels ensured a larger dataset for the study analysis. These results also suggest that VI assays may be evaluated with the less stringent IC50 rather than the usual IC90.

Comparison of the two types of assays, the PSV assay and PBMC, with TriMab, a mixture of three monoclonal antibodies, indicated that the PSV assay detects HIV-1 neutralization with higher sensitivity than PBMC (p = 0.014). This relationship was confirmed over time and was similar in both Phase I and II studies. However, a clear difference in sensitivities of the two types of assays could not be established with polyclonal reagents. When neutralizing activity was examined across viruses or across plasma the two assay types showed comparable sensitivities for most but not all combinations. PSV appeared to be more sensitive in some but not all virus-plasma combinations and sensitivity was dependent on both the virus and the plasma. We cannot exclude that the apparent higher sensitivity of the PSV assay with some virus-plasma combinations as compared to VIA could be due to the pre-selection of the polyclonal reagents done with PSV assays. Also, polyclonal samples containing different Ig subtypes with various functional activity, together with PBMC, a mix of varieties of cell types responsible for different inhibitory functions, may exert a differential effect on replication of the different viruses. Specificities of the plasma used in our study were not tested and reactivity might be against multiple epitopes present. Furthermore, it is clear that for cases where specificity testing was attempted, it was concluded that reactivity against multiple epitopes was present, some of which are well known, while others are not typed yet [37], [38], [39].

Results were markedly different with the 92UG024 virus, being extremely sensitive (Tier 1) in the PSV assay but showing a more resistant profile in VIA. However assay results of the four laboratories performing the PSV assay were very disparate. Interestingly the nature of the virus, single- versus multiple-round infection might have had a dramatic impact for this particular virus-plasma combination. However, such dramatic differences were not seen with the other inhibitory reagents used. We can also not exclude that the amino acid differences (n = 5) seen between the culture supernatant and the PSV plasmid might have been responsible for the observed differences.

Due to the set-up of our NeutNet study each lab was obliged to use their own protocol both for virus production and neutralization assay. This is different to the comparative study by Todd et al. [16] in which the goal was to compare neutralization results obtained by several laboratories all using the same assay (PSV-TZMbl). From that study the authors concluded that pseudovirus stocks generated in individual laboratories were a major source for assay variability. Inter-laboratory results were more homogenous when the same titrated PSV stocks were distributed among participants. In our study, HEK293T cells were from a common source, whereas production and titration of PSV stocks were done in individual laboratories. This is likely to have contributed to assay variability, which however was anyhow limited. In addition, our study aimed at comparing different protocols rather than use of one standard protocol.

Within the group of VIAs, the conventional PBMC assay was compared to the PR assay using the GHOST(3) cell line. The previously standardized and validated PR assay has been subjected to automated reading allowing high-throughput application and further improvement of assay sensitivity. In particular, the high-throughput readout gives results that are within the range of variation of PBMC assays. Since the PBMC assay is cumbersome and difficult to standardize, the use of the reliable, simple and cheap PR assay is encouraged.

However, the recommendation of one assay for general use is complicated by the fact that we still lack knowledge about which *in vitro* assay best correlates with *in vivo* protection. The variation of assay sensitivity with the virus and the inhibitory reagent justifies the use of both types of assays, PSV and VIA. Although initially our goal was to choose “the best” assay for use in vaccine research and clinical vaccine trials, the most important lesson learned is that no assay alone detects neutralization over the entire spectrum of virus-reagent combinations [17], [40]. In addition to neutralization, additional inhibitory activity of antibodies, identified as antibody-dependent cellular virus inhibition (ADCVI) or antibody-dependent cellular cytotoxicity (ADCC), may contribute to HIV protection [41]. For ADCC and ADCVI different protocols exist [42], [43], and possibly a similar exercise as the one performed within NeutNet should be considered for other functional assays.

Future work should aim at clarifying the biological significance of both neutralizing and non-neutralizing antibodies detected in the different assays. If a correlation between *in vitro* antibody mediated viral inhibition and *in vivo* protection can be established, it will also be possible to choose the most appropriate assay to measure such antibodies in future vaccine trials.

In summary, clear differences in assay sensitivities, dependent on both the neutralizing reagent and the virus, were once again demonstrated. As previously, the use of both PSV and VI neutralization assays are recommended for vaccine evaluation.

## Supporting Information

Figure S1 Mean inhibitory concentration (IC) 50 values for duplicate assays performed with HIV negative plasma (ARP523) and virus as indicated. The cells are colour coded: green, poor or no neutralization, reciprocal plasma dilution <20; yellow, reciprocal plasma dilution 20–160. Assays are grouped as in Figure 2. Laboratories performing the assays are numbered and colour coded.
(TIF)

Figure S2 Partial amino acid sequence alignment of 92UG024 from culture supernatant and PSV plasmid. Differences in sequence were highlighted.
(DOCX)

Figure S3 Inhibitory concentration (IC) 50 values generated by laboratories using 92UG024 PSV, using either plasmid (6A, 10 and 13) or culture supernatant (12) as starting material for virus production, as compared to IC50’s of PBMC using laboratories.
(TIF)

Table S1 Selection and characterization of plasma samples. (A) Characterization of samples obtained from Zeptometrix. (B) and (C) Selection of blood donor samples obtained through NIBSC. Foot note: Colour code for assays: orange, indicates IC50 in the PSV (DNA) - TZMbl assay; minimum assay cut off <20. Pink, indicates IC50 in the PSV recombinant virus assay (CC), except for *(DNA used; CC not tested); blue, titer given in percentage using a 1: 30 plasma dilution (S1A) or in IC90 (S1C) in the PBMC assay (mean of 2 tests). Minimum assay cut off or negative values in white. Selected plasma samples are in bold (in Table S1B and S1C). Results are displayed for viruses tested simultaneously in two or three assays. Another 13 and 10 viruses were tested in the PSV-TZMbl and PSV recombinant assay, respectively (S1B and S1C).
(XLSX)

Table S2 Intra-laboratory consistency of inhibitory concentrations (IC). Foot note: (S2a); Analysis was restricted to tests where it had been possible to calculate an IC value from the data for both tests, and the calculated IC values were within the dilution range used (20–1280). Values were calculated in two ways (1) individually for each IC value across repeat tests that satisfied the predefined criteria and (2) restricted to tests where all three IC values could be calculated. (S2b); Based on data from laboratories 2, 4B, 6A, 10, 12 and 13 (PSV) and laboratories 3B, 6B, 7, 8, 14 and 15 (PBMC). A two-sample t-test was used on the pooled set of fold differences to compare the PSV and PBMC consistency.
(XLSX)

Table S3 Inter-laboratory comparisons. Foot note: For each laboratory, a geometric mean IC value for the repeat tests was calculated. For each virus and inhibitory combination, an overall geometric mean of the individual laboratory means was calculated, along with the minimum, maximum, and range between laboratories. Values of the ICs are expressed as µg/ml for TriMab and as reciprocal dilutions for plasma. To allow calculations of the geometric means, any IC value that was greater than the highest dilution used were taken as equal to the next two-fold dilution step, so results recorded as >1280 were taken as equal to 2560. Similarly, IC values that were below the lowest dilution were taken as the next two-fold dilution step (e.g. <20 was converted to 10). To calculate fold-ranges for inter-laboratory comparisons, a conservative estimate was calculated by taking, for example <20 = 20, to give a minimum fold-range. N lab, number of laboratories involved.
(XLSX)
