# Supplementary figures and images for: International Network for Comparison of HIV Neutralization Assays: The NeutNet Report II
Source: PLoS One. 2012 May 9;7(5):e36438. doi: 10.1371/journal.pone.0036438 (PMC3348930; doi:10.1371/journal.pone.0036438)

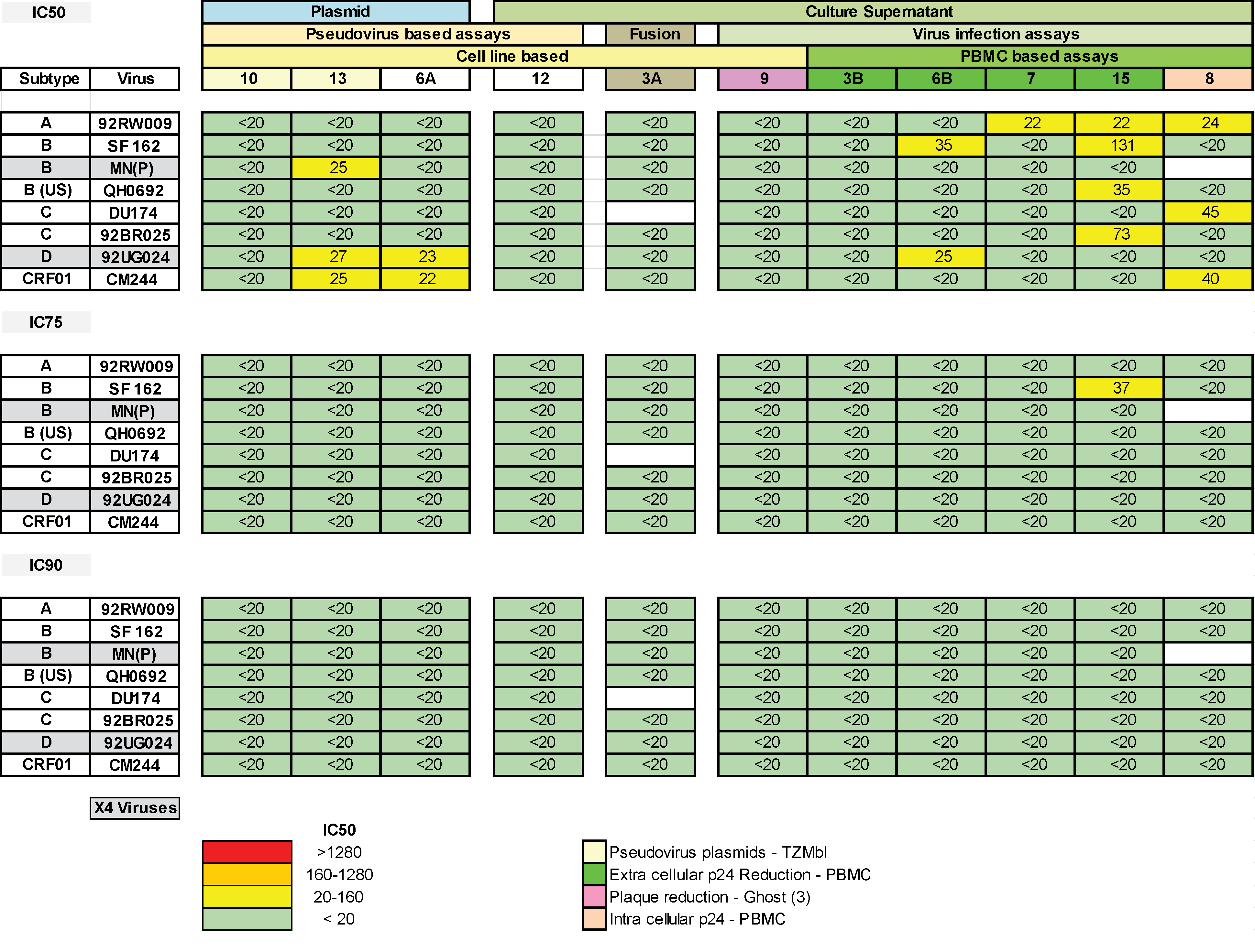

Supplement: Figure S1 — Mean inhibitory concentration (IC) 50 values for duplicate assays performed with HIV negative plasma (ARP523) and virus as indicated. The cells are colour coded: green, poor or no neutralization, reciprocal plasma dilution <20; yellow, reciprocal plasma dilution 20–160. Assays are grouped as in Figure 2. Laboratories performing the assays are numbered and colour coded. (TIF) [file pone.0036438.s001.tif]

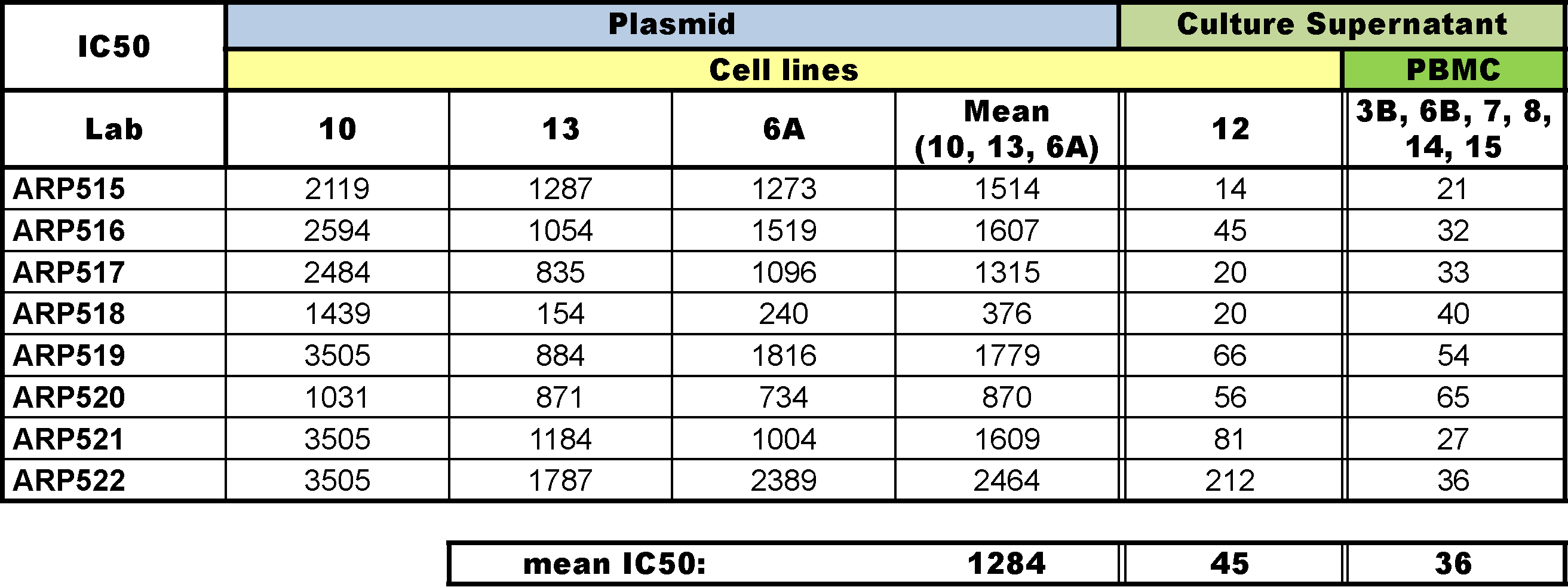

Supplement: Figure S3 — Inhibitory concentration (IC) 50 values generated by laboratories using 92UG024 PSV, using either plasmid (6A, 10 and 13) or culture supernatant (12) as starting material for virus production, as compared to IC50’s of PBMC using laboratories. (TIF) [file pone.0036438.s003.tif]
