# Supplementary material for: International Network for Comparison of HIV Neutralization Assays: The NeutNet Report II
Source: PLoS One. 2012 May 9;7(5):e36438. doi: 10.1371/journal.pone.0036438 (PMC3348930; doi:10.1371/journal.pone.0036438)
Supplement: Figure S2 — Partial amino acid sequence alignment of 92UG024 from culture supernatant and PSV plasmid. Differences in sequence were highlighted. (DOCX) [file pone.0036438.s002.docx]

10 20 30 40 50 60 70 80 90 100

....|....|....|....|....|....|....|....|....|....|....|....|....|....|....|....|....|....|....|....|

**HxB2**  **MRVKEKYQHLWRWGWRWGTMLLGMLMICSATEKLWVTVYYGVPVWKEATTTLFCASDAKAYDTEVHNVWATHACVPTDPNPQEVVLVNVTENFNMWKNDM**

**UG024SN** **----------------------------------------------------------------------------------------------------**

**UG024pv** **----------------------------------------------------------------------------------------------------**

110 120 130 140 150 160 170 180 190

....|....|....|....|....|....|....|....|....|....|....|....|....|....|....|....|....|--....|....|...

**HxB2**  **VEQMHEDIISLWDQSLKPCVKLTPLCVSLKCTDLKNDTNTNSSSGRMIMEKGEIKNCSFNISTSIRGKVQKEYAFFYKLDIIPID--NDTTSYKLTSCNT**

**UG024SN** **----------------------TPLCVTLNCIEWKNENETKGNKTVDPDKDIGMKNCSFNVTTEVRDKKKQVYALFYKLDVVQMNDNNTNTSYRLINCNT**

**UG024pv** **----------------------TPLCVTLNCIEWKNENETKGNKTVDPDKDIGMKNCSFNVTTEVRDKKKQVYALFYKLDVVQMNDNNTNTSYRLINCNT**

200 210 220 230 240 250 260 270 280 290

.|....|....|....|....|....|....|....|....|....|....|....|....|....|....|....|....|....|....|....|...

**HxB2**  **SVITQACPKVSFEPIPIHYCAPAGFAILKCNNKTFNGTGPCTNVSTVQCTHGIRPVVSTQLLLNGSLAEEEVVIRSVNFTDNAKTIIVQLNTSVEINCTR**

**UG024SN** **SAITQACPKVTFEPIPIHYCAPAGFAILKCNNKMFNGTGPCNNVSTVQCTHGIRPVVSTQLLLNGSLAEEEIIIRSENITNNAKIIIVHLNESVVINCTR**

**UG024pv** **SAITQECPKVTFEPIPIHYCAPAGFAILKCNNKMFNGTGPCNNVSTVQCTHGIRPVVSTQLLLNGSLAEEEIIIRSENITNNAKIIIVHLNESVVINCTR**

300 310 320 330 340 350 360 370 380 390

.|....|....|....|....|....|....|....|....|....|....|....|....|....|....|....|....|....|....|..-..|..

**HxB2**  **PNNNTRKRIRIQRGPGRAFVTIGKIGNMRQAHCNISRAKWNNTLKQIASKLREQFGNNKTIIFKQSSGGDPEIVTHSFNCGGEFFYCNSTQLFN-STWFN**

**UG024SN** **PYNNIRQRTPI--GLGQALYTTRRIEDIRRAHCNISEAAWKKTLEQVAKKLKVLF-NRTTIDFKPSSGGDPEITTHSFNCGGEFFYCNTSKLFNHSVGEN**

**UG024pv** **PYNNIRQRTPI--GLGQALYTTRRIEDIRRAHCNISEAAWKKTLEQVAKKLKVLF-NRTTIDFKPSSGGDPEITTHSFNCGGEFFYCNTSKLFNHSVGEN**

400 410 420 430 440 450 460 470 480 490

..|....|....|....|....|....|....|....|....|....|....|....|....|....|....|....|....|....|....|....|..

**HxB2**  **STWSTEGSNNTEGSDTITLPCRIKQIINMWQKVGKAMYAPPISGQIRCSSNITGLLLTRDGGNSNNESEIFRPGGGDMRDNWRSELYKYKVVKIEPLGVA**

**UG024SN** **NTLSISNGVNTN----ITLPCKIKQIVNMWQGVGKAMYAPPIQGLIRCSSNITGLLLTRDGGNTS-QNETFRPGGGDMRDNWRSELYKYKVIKIEPLGLA**

**UG024pv** **NTLSISNGVNTN----ITLPCKIKQIVNMWQGVGKAMYAPPIQGLIRCSSNITGLLLTRDGGNTS-QNETFRPGGGDMRDNWRSELYKYKVIKIEPLGLA**

500 510 520 530 540 550 560 570 580 590

..|....|....|....|....|....|....|....|....|....|....|....|....|....|....|....|....|....|....|....|..

**HxB2**  **PTKAKRRVVQREKRAVGIGALFLGFLGAAGSTMGAASMTLTVQARQLLSGIVQQQNNLLRAIEAQQHLLQLTVWGIKQLQARILAVERYLKDQQLLGIWG**

**UG024SN** **PTRAKRRVVAREKRAIGLGAVFLGFLGAAGSTMGAASLTLTVQARQLMSGIVQQQNNLLRAIEAQQHLLQLTVWGIKQLQARVLAVESYLKDQQLLGXWG**

**UG024pv** **PTRAKRRVVAREKRAIGLGAVFLGFLGAAGSTMGAASLTLTVQARQLMSGIVQQQNNLLRAIEAQQHLLQLTVWGIKQLQARVLAVESYLKDQQLLGVWG**

600 610 620 630 640 650 660 670 680 690

..|....|....|....|....|....|....|....|....|....|....|....|....|....|....|....|....|....|....|....|..

**HxB2**  **CSGKLICTTAVPWNASWSNKSLEQIWNHTTWMEWDREINNYTSLIHSLIEESQNQQEKNEQELLELDKWASLWNWFNITNWLWYIKLFIMIVGGLVGLRI**

**UG024SN** **CSGRHICPTRVPWNSSWSNKSLDEIWGNMTWMEWEREISNYTGLIYNLIEESQIQQEKNEKDLLELDKWASLWNWFDITNWLWYIRIFIIIVGSLIGLRI**

**UG024pv** **CSGRHICPTRVPWNSSWSNKSLDEIWGNMTWMEWEREISNYTGLIYNLIEESQIQQEKNEKDLLELDKWASLWNWFDITNWLWYIRIFIIIVGSLIGLRI**

700 710 720 730 740 750 760 770 780 790

..|....|....|....|....|....|....|....|....|....|....|....|....|....|....|....|....|....|....|....|..

**HxB2**  **VFAVLSIVNRVRQGYSPLSFQTHLPTPRGPDRPEGIEEEGGERDRDRSIRLVNGSLALIWDDLRSLCLFSYHRLRDLLLIVTRIVELLGRRGWEALKYWW**

**UG024SN** **VFAVLSLVNRVRQGYSPLSFQTLLPTPRGPDRPGXTXEEGGEQGRGRSIRLLNGLSALIWDDLRNLCLFSYHHLRDLILIAARIVGLLGRRGWEAIKYLW**

**UG024pv** **VFAVLSLVNRVRQGYSPLSFQTLLPTPRGPDRPGETEEEGGEQGRGRSIRLLNGLSALIWDDLRNLCLFSYHHLRDLILIAARIVGLLGRRGWEAIKYLW**

800 810 820 830 840 850

..|....|....|....|....|....|....|....|....|....|....|......

**HxB2**  **NLLQYWSQELKNSAVSLLNATAIAVAEGTDRVIEVVQGACRAIRHIPRRIRQGLERILL**

**UG024SN** **NLLQYWIQELKNSAIXLFNTTAIVVAEGTDRIIELIQRIGRAILNIPTRIRQGLERLLL**

**UG024pv** **NLLQYWIQELKNSAISLFNTTAIVVAEGTDRIIELIQRIGRAILNIPARIRQGLERLLL**

**Legend**:

Partial sequence comparison (2190 bp), the NH2-terminal part (122 amino acids) was not sequenced:

UG024sup: Sequence derived from 92UG024 virus culture supernatant (ARP177.4)

UG024pv: Sequence derived from pSVIII-92UG024.2 cl 2 plasmid (ARP239.13)

Both sequence differences between supernatant and plasmid as well as mixtures of amino acids are highlighted.

*Amino acid numbering according to HxB2 (<http://www.hiv.lanl.gov/content/sequence/HIV/REVIEWS/HXB2.html>)
